# Correlation between retinal function and microstructural foveal changes in intermediate age related macular degeneration

**DOI:** 10.1186/s40942-017-0061-3

**Published:** 2017-05-08

**Authors:** Serena Fragiotta, Carmela Carnevale, Alessandro Cutini, Enzo Maria Vingolo

**Affiliations:** Department of Medical–Surgical Sciences and Biotechnologies, U.O.C. Ophthalmology, Sapienza University of Rome, Via Firenze, 04019 Terracina, Italy

**Keywords:** Intermediate age related macular degeneration, Microperimetry, Bivariate contour ellipse area, Retinal sensitivity, Spectral domain optical coherence tomography

## Abstract

**Purpose:**

To assess foveal microstructural changes influencing retinal sensitivity (RS) and fixation stability using microperimeter MP-1 in intermediate age-related macular degeneration (AMD).

**Methods:**

In this cross-sectional study, 22 eyes of 22 patients (mean age: 75 ± 9.02 years) with intermediate AMD were enrolled. Retinal sensitivity and bivariate contour ellipse area (BCEA) were obtained by microperimetry MP-1 (Humphrey 10-2 68-loci grid) under mesopic conditions. Drusen type, drusenoid pigment epithelial detachment, hyperreflective foci (HF), integrity of external limiting membrane (ELM), inner ellipsoid zone (ISel), RPE/Bruch’s membrane complex (RPE/B) and subfoveal choroidal thickness were analyzed in the foveal region and compared with RS and BCEA. Spearman’s rank correlation coefficient was used to evaluate the relationship between variables. Logistic regression analysis was also used to assess morphological predictor influencing RS or BCEA.

**Results:**

RS was strongly and inversely related with the presence of HF (r = −0.66, P = 0.001), integrity of ELM (r = −0.70, P < 0.001), ellipsoid zone (r = −0.45, P = 0.03). Instead, BCEA is positively related to the ellipsoid zone integrity (r = 0.45, P = 0.03). Logistic regression analysis confirmed that disruption of ISel influenced fixation stability (ExpB: 9.69, P = 0.04) but not RS. Instead, the presence of HF and disruption of ELM predicted RS reduction (ExpB: 0.55, P = 0.02 and ExpB: 0.29, P = 0.04, respectively).

**Conclusions:**

The integrity of ELM and the presence of HF are both predictors of RS. The ELM status may be considered a new biomarker of retinal function together with HF. Instead, the integrity of ISel band seems to be a more selective predictor of BCEA than RS.

**Electronic supplementary material:**

The online version of this article (doi:10.1186/s40942-017-0061-3) contains supplementary material, which is available to authorized users.

## Background

Large confluent drusen and macular hyperpigmentary changes in intermediate age-related macular degeneration (AMD) are well known risk factors for progression to geographic atrophy (GA) and choroidal neovascularization (CNV) [1–3].

Spectral-domain optical coherence tomography (SD-OCT) allows a high definition cross-sectional visualization of microstructural alterations providing a more accurate detection of biomarkers for AMD progression [4, 5]. Microstructural changes investigated in intermediate AMD as possible biomarker of advanced disease included presence of hyperreflective foci (HF), inner-segment ellipsoid zone integrity (ISel), drusen area, drusenoid pigment epithelial detachment (DPED), and retinal pigment epithelium (RPE) integrity [4–9].

Microperimetry has been considered a useful functional test in AMD even superior to conventional visual acuity [10–13]. Some microstructural alterations have been related to retinal sensitivity (RS) decline even in intermediate AMD patients [14–16].

Fixation stability is a functional parameter directly related with both visual acuity and RS that allows a more accurate visual function evaluation. In fact, it has been reported that patients with advanced AMD have impaired fixation stability and this may contribute to their poor visual performance [17–19].

However, no study to date has examined the association between microstructural changes and fixation stability in intermediate AMD. Accordingly, RS and fixation stability have never been included together in a statistical model to determine their respective morphological predictors. Moreover, no other studies considered the relation between the integrity of all three outer retinal bands (ELM, ISel, and RPE) and functional parameters. Therefore, the purpose of this study was to assess the influence of microstructural alterations by high-resolution SD-OCT imaging on RS and fixation stability.

## Methods

In this cross-sectional study, 22 eyes of 22 patients (13 female and 9 male) aged 50 years or older with intermediate AMD (mean age, 75 ± 9.02 years) were enrolled. The criteria for including intermediate AMD eyes were drusen 125 μm or larger, or drusen of 63 μm or larger but smaller than 125 μm and pigmentary abnormalities according to the Beckmann classification [20]. Institutional review board (IRB) permission was granted. All procedures adhered to the tenets of the Declaration of Helsinki. Each participant gave informed consent prior to enrollment in the study.

Exclusion criteria included the presence of any choroidal neovascularization or geographic atrophy, significant concomitant ocular pathologies such as media opacities, history of vitreoretinal surgery or laser therapy.

All patients underwent a complete ophthalmic examination, including Snellen visual acuity testing, slit-lamp examination, fundus examination, retinography, SD-OCT, and fluorescein angiography and/or indocyanine green angiography as needed. Snellen acuity was converted into logarithm of the minimum angle of resolution for statistical analysis.

### Imaging

**Fig. 1:**
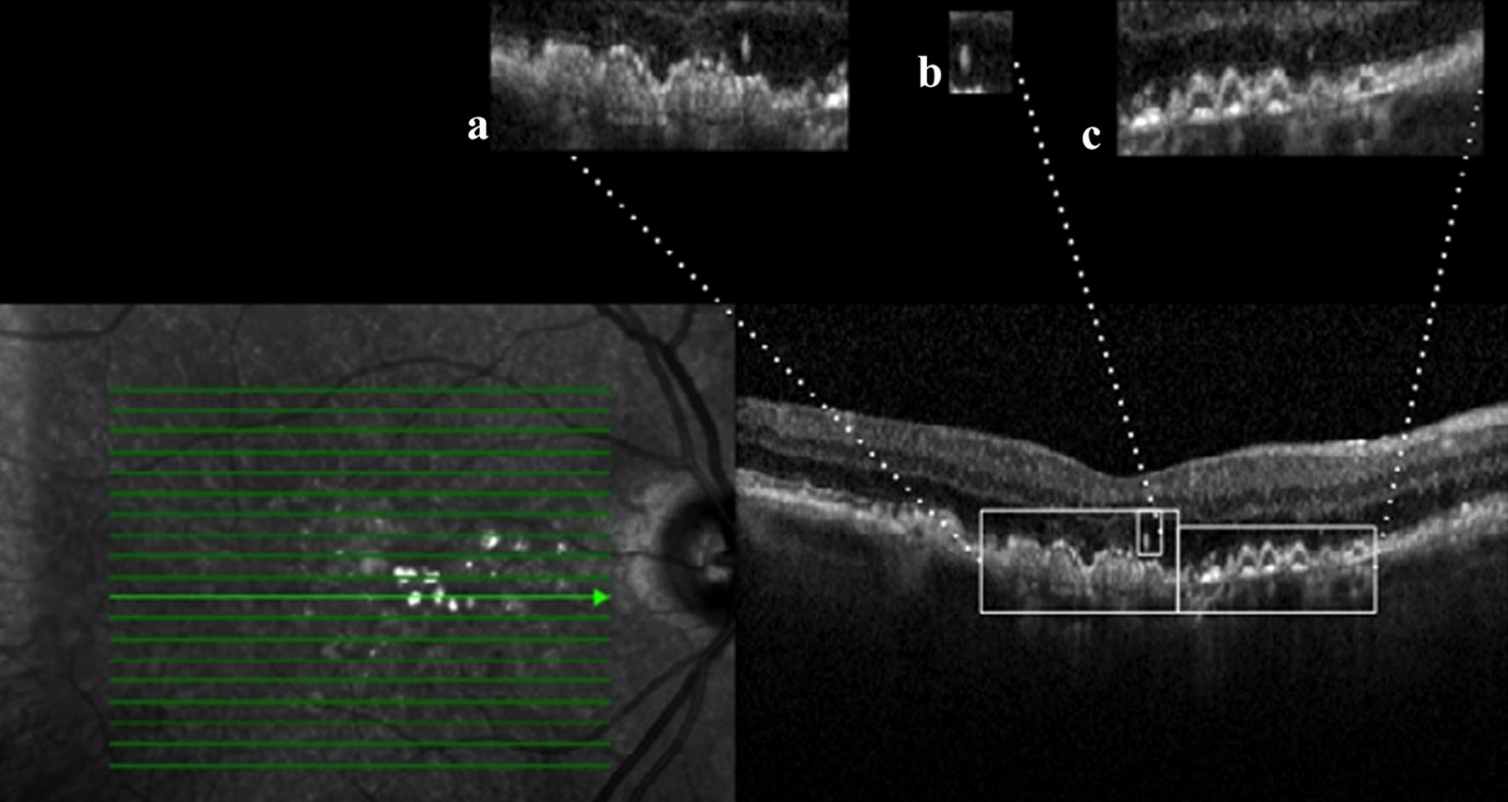
Composition of the different SD-OCT phenotypes analyzed. *A* presence of drusenoid pigment epithelial detachment (DPED); *B* presence of hyperreflective foci (HF); *C* lack of integrity external limiting membrane (ELM), inner-segment ellipsoid band integrity (ISel), and retinal pigment epithelium (RPE)

All the SD-OCT scans were obtained using the Heidelberg Spectralis SD-OCT (software version 5.4.7.0; Heidelberg Engineering). OCT volumes were acquired across a 20° × 15° (5.8 × 4.3 mm) rectangle centered on the fovea. Twenty-five line scans were acquired with Automatic real time (ART) mode sets at 50 frames. Quality score with signal-to-noise 20 dB or above was obtained before scans acquisition. Central macular thickness (CMT) was measured automatically with OCT mapping software. Enhanced depth imaging (EDI) mode was selected to improve visualization of the choroid. The following parameters were collected: (I) types of drusen: (a) conventional (hard/soft); (b) reticular pseudodrusen; (II) Condition of (a) ELM; (b) ISel; (c) RPE; (III) presence/absence of HF; (IV) presence/absence of DPED; (V) subfoveal choroidal thickness (SCT). Figure 1 summarized SD-OCT phenotypes analyzed.

Drusenoid pigment epithelial detachment is defined following AREDS criteria as a well-defined, pale yellow or white, large mound consisting of many large drusen or confluent drusen that is at least 350 μm in the narrowest diameter [5]. SD-OCT anatomical landmarks according to IN*OCT consensus were pursued in order to distinguish outer retinal layers. The innermost discrete hyper-reflective band at the outer border of the outer nuclear layer, and above the ellipsoid zone was considered as external limiting membrane; the hyperreflective band underneath the hyperreflective ELM and the hyporeflective myoid zone was referred as ellipsoid zone; the outermost hyperreflective band was defined as RPE/Bruch’s complex [21]. The interdigitation zone (first hyper-reflective band above the RPE) was excluded by analysis because it is not always recognizable in the SD-OCT scans, even in healthy eyes [22, 23].

### Microperimetry examination

Microperimetry was performed using the MP-1 microperimeter (NAVIS, software version 1.7.6; Nidek Technologies, Japan). The examination was conducted by a single experienced examiner (CC) under mesopic condition and pupils were dilated with 1 drop each of tropicamide 1% and phenylephrine 2.5%. Each subject was instructed to maintain fixation on the central target and to press a push-button if any light stimulus was seen.

A red cross of 2° and 1 unit thickness (10 minarc) was used as a fixation target for both preliminary fixation test and microperimetry examination. MP was performed used white background illumination of 4 asb (1.27 cd/m^2^) and stimulus size Goldmann III, with a projection time of 200 ms. A 68-loci customized grid covering the central 20°, centered on the fovea was select as pattern. We used a 4-to-2-staircase strategy and the initial projecting sensitivity was fixed at 16 dB. Patients underwent a brief training at the beginning of each MP and “Pre-test” option was selected. Spherical error was manually typed into the window before starting the examination.

**Fig. 2:**
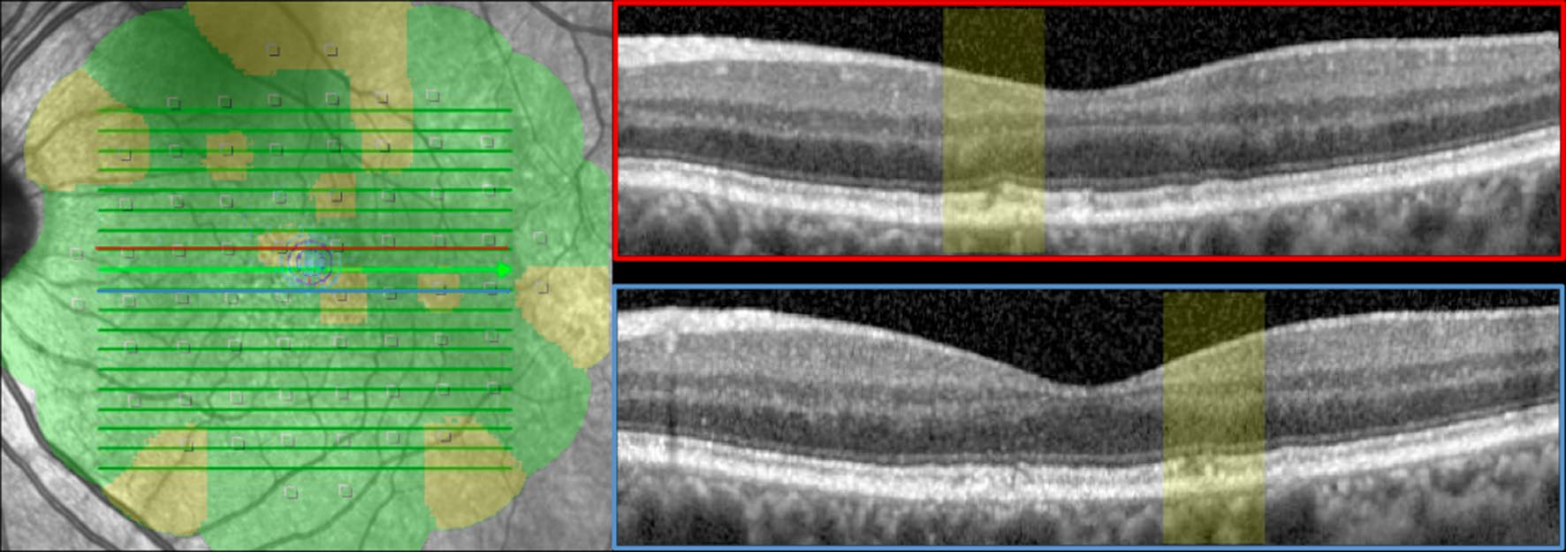
Interpolation between microperimetric map and SD-OCT scans. Microperimetric map and BCEA area were superimposed onto infrared image. Relative sensitivity defects correspond to area of ellipsoid band discontinuity within two foveal scans (marked as *pale blue* and *red*)

Stability of fixation was quantified using bivariate contour ellipse area (BCEA) and the formula previously described by Timberlake [24]. The BCEA encompassing 68% of fixation points (±1 standard deviation) was considered for statistical analysis (see also Fig. 2).

### Data analysis

It was evaluated microstructure of the central fovea × 1.39 mm^2^, consisting of 5 consecutive horizontal B-scans. Therefore resulting in an area of 0.97 × 1.43 mm^2^ centered over the fovea. Drusen type, DPED, HF, integrity of ELM, ISel, RPE and subfoveal CT were analyzed in the foveal region and compared with RS and BCEA.

The condition of those bands was classified as ‘continuous’ whenever they were clearly detectable and without interruption. The bands were defined as ‘discontinuous’ if they appeared blurred and/or interrupted. Two experienced ophthalmologists (SF, AC) independently interpreted the SD-OCT scans masked to the other clinical findings; disagreement between readers was resolved by senior retinal specialist (EMV).

Microperimetric software automatically calculates RS map and BCEA values, mean RS was obtained manually drawing a polygon enclosing all the RS points.

The normality of distributions was verified by Shapiro–Wilk normality test. BCEA (deg^2^) was normalized by logarithmic transformation when necessary (Shapiro–Wilk test, P < 0.05). Spearman’s rank correlation coefficient was used to evaluate the relationship between variables. Binary logistic regression analysis was also used to assess morphological predictor influencing RS or BCEA. P value less than 0.05 were considered statistically significant, and P < 0.001 was considered to be highly statistically significant. All calculations were carried out using the SPSS software (ver. 20; SPSS, Inc., Chicago, IL).

## Results

Main characteristics from the participants involved in our cross-sectional study are summarized in Table 1. Drusen sub-classification revealed soft drusen in 12 eyes (54.54%) reticular pseudodrusen in 9 eyes (40.91%), and cuticular drusen in 1 eye (4.54%).

**Table 1:** Main characteristics of intermediate age-related macular degeneration patients

	Eyes (n = 22)
Age (years)	75 ± 9.02
Retinal sensitivity (dB)	12.67 ± 2.34
BCEA, deg^2^ (95% CI)	1.94 (0.90, 2.98)
logMAR, mean (95% CI)	0.07 (0.002, 0.15)
Presence of HF, n (%)	10 (45.45%)
Continuity of ELM, n (%)	8 (36.36%)
Continuity of ISel, n (%)	6 (27.27%)
Continuity of RPE, n (%)	6 (27.27%)
Presence of DPED, n (%)	14 (63.63%)
Subfoveal CT	188.86 ± 62.29

All data are expressed as mean ± SD if not otherwise specified

*BCEA* bivariate contour ellipse area, *logMAR* logarithm of the minimum angle of resolution, *CI* confidence interval, *HF* hyperreflective foci, *ELM* external limiting membrane, *ISel* inner ellipsoid zone, *RPE* retinal pigment epithelium, *DPED* drusenoid pigment epithelial, *CT* choroidal thickness

LogMAR acuity was significantly correlated with RS (r = −0.50, P = 0.02). Moreover, a positive significant relationship was found with ellipsoid zone as well as RPE integrity (r = 0.49, P = 0.02 in both cases).

The RS was strongly and inversely related with the presence of HF (r = −0.66, P = 0.001) and integrity of ELM (r = −0.70, P < 0.001) but moderate correlated with ellipsoid zone integrity (r = −0.45, P = 0.03). Instead, BCEA is positively related to the ellipsoid zone integrity (r = 0.45, P = 0.03). No relationships were found between drusen type and RS (r = −0.33, P = 0.12) or BCEA (r = −0.17, P = 0.45).

Subfoveal CT was inversely related with drusen type (r = −0.44, P = 0.03), age (r = −0.50, P = 0.02) and ELM integrity (r = −0.51, P = 0.01) but no with functional parameters. Moreover, the presence of HF was strongly related with ELM integrity (r = 0.83, P < 0.001), but also with ISel and RPE bands (r = 0.56, P < 0.001), and the presence of DPED (r = 0.45, P = 0.03).

Logistic regression analysis confirmed that disruption of ISel influenced fixation stability (ExpB: 9.69, P = 0.04) but not RS. Instead, the presence of HF and discontinuity of ELM predicted RS decrease (ExpB: 0.55, P = 0.02 and ExpB: 0.29, P = 0.04, respectively).

## Discussion

In this study we compared microstructural changes with RS and fixation stability using microperimetry. Our findings are consistent with previous studies indicating that RS is strictly related to the integrity of ISel band and the presence of HF, but none of these observations have evaluated the ELM [9, 14, 16, 25]. Interestingly, in our study, RS showed the strongest correlation with ELM discontinuity. Moreover, fixation stability analysis adds further interesting information, resulting affected by outer retinal bands integrity as well.

Larger drusen may become confluent and are often accompanied by disruption of the ellipsoid zone and ELM [6]. The status of ELM has been associated with visual acuity (VA), it was more highly related to VA than was the ellipsoid zone [26]. Not surprisingly, ELM status has been reported as a good indicator for visual prognosis in eyes with neovascular AMD, confirming that the ELM was directly correlated with final VA whereas ellipsoid zone did not [27, 28]. Interestingly, in our study the status of ELM was the strongest predictor of RS. Possibly this is because the integrity of the ELM has a critical role in restoration of the photoreceptor microstructure and alignment [29–31]. We also noted a direct relation between HF and ELM status; this may be related to the ability of RPE to migrate into outer nuclear layers. However a coordinate opening of ELM during RPE migration remains to be determined [32].

Although both BCEA and RS were significantly correlated with ellipsoid zone integrity; by combining microstructural factors in a logistic regression model to predict BCEA and RS, the discontinuity of ISel band predicts BCEA enlargement but not the RS. These findings are partially in contrast with previous reports that considered RS as the strongest predictor of ellipsoid zone integrity [14, 16, 25]. Such discrepancy can be easily explained by the fact that BCEA was never analyzed before in a similar model, thus it may serve as more selective biomarker of photoreceptor damage than RS. Conversely in stages not involving photoreceptor band, RS is superior to BCEA in detecting functional dysfunction.

The BCEA is a quantitative functional parameters directly related with both visual acuity and RS that allows a more accurate quantification in visual function, especially reading speed [17, 19, 33]. It has been reported that BCEA decline progressively during geographic atrophy progression [34]. Moreover, patients with scotoma usually develop a discrete of eccentric fixation called preferred retinal locus (PRL) and also an increase in fixation stability area, contributing to a poor visual performance [17–19, 33]. Accordingly, we may speculate that fixation stability may be early affected in AMD even if a significant scotoma is not detected.

In summary, this study confirms that AMD can formerly cause visual dysfunction in the intermediate stages. Further, our findings provide additional morpho-functional information that deserves further investigations. ELM band status is strongly associated with RS and it may represent a new predictive factor of retinal function together with previously known hyperreflective foci. Moreover, BCEA seems to be a selective biomarker of photoreceptor damage greater than RS.

 Various limitations of this study must be considered. First, our study included a small sample size. Second, morphological analysis is limited to the foveal region and it does not provide any point-to-point correlation. Despite these limitations, our goal was to understand which morphological factors influence RS but especially fixation stability that is essentially a foveal function. Further studies will be needed to understand if functional damage may be an early predictor of neovascular conversion. It may help to identify high-risk patients who may benefit of early therapeutic and rehabilitative strategies.

## Additional file

**Additional file 1.** Study dataset.
